# *Plasmodium falciparum *population dynamics in a cohort of pregnant women in Senegal

**DOI:** 10.1186/1475-2875-9-165

**Published:** 2010-06-16

**Authors:** Juliette Guitard, Pernille Andersen, Caroline Ermont, Sédami Gnidehou, Nadine Fievet, Ole Lund, Philippe Deloron, Nicaise Tuikue Ndam

**Affiliations:** 1 UR010, Institut de Recherche pour le Développement (IRD), Université Paris Descartes, 75006, France; 2 Centre for Medical Parasitology, Department of International Health, Immunology and Microbiology, University of Copenhagen, Denmark; 3 Center for Biological Sequence Analysis, Department of Systems Biology, Technical University of Denmark. 2800 Lyngby, Denmark

## Abstract

**Background:**

Pregnant women acquire protective antibodies that cross-react with geographically diverse placental *Plasmodium falciparum *isolates, suggesting that surface molecules expressed on infected erythrocytes by pregnancy-associated malaria (PAM) parasites have conserved epitopes and, that designing a PAM vaccine may be envisaged. VAR2CSA is the main candidate for a pregnancy malaria vaccine, but vaccine development may be complicated by its sequence polymorphism.

**Methods:**

The dynamics of *P. falciparum *genotypes during pregnancy in 32 women in relation to VAR2CSA polymorphism and immunity was determined. The polymorphism of the *msp2 *gene and five microsatellites was analysed in consecutive parasite isolates, and the *DBL5ε + Interdomain 5 *(*Id5*) part of the *var2csa *gene of the corresponding samples was cloned and sequenced to measure variation.

**Results:**

In primigravidae, the multiplicity of infection in the placenta was associated with occurrence of low birth weight babies. Some parasite genotypes were able to persist over several weeks and, still be present in the placenta at delivery particularly when the host anti-VAR2CSA antibody level was low. Comparison of diversity among genotyping markers confirmed that some PAM parasites may harbour more than one *var2csa *gene copy in their genome.

**Conclusions:**

Host immunity to VAR2CSA influences the parasite dynamics during pregnancy, suggesting that the acquisition of protective immunity requires pre-exposure to a limited number of parasite variants. Presence of highly conserved residues in surface-exposed areas of the VAR2CSA immunodominant DBL5ε domain, suggest its potential in inducing antibodies with broad reactivity.

## Background

In sub-Saharan Africa, 25 million pregnant women are at risk of *Plasmodium falciparum *infection every year[1,2]. *Plasmodium falciparum *isolates infecting pregnant women are special in that parasites are able to sequester *in vivo *in the intervillous space and bind *in vitro *to chondroitin sulphate A (CSA) [3,4]. This placental accumulation of infected red blood cells (IRBCs) is associated with the low birth weight of the newborn [5].

The parasite ligand involved in this interaction between the IRBCs and CSA is a specific PfEMP1 variant named VAR2CSA [6-8]. Serum samples from exposed pregnant women specifically recognize CSA-binding infected erythrocytes, independently of their geographic origin and are able to inhibit binding to CSA *in vitro*. This suggests that the antigenic targets specifying placental parasites must be relatively conserved [9,10]. Protection against pregnancy-associated malaria (PAM) is rapidly acquired and is associated to high level of anti-VAR2CSA antibodies. Thus, in areas of high *Plasmodium *transmission, primigravid women lack those antibodies and are the most at risk of developing severe consequences of PAM [11-16].

All this highlights the key role that VAR2CSA plays in the mechanism of PAM and the acquisition of protective immunity and clearly opens an excellent opportunity for the development of a vaccine against PAM [17]. However, the problem of polymorphism accounting for 10-30% variation between the different clinical isolates [18,19] represents a great challenge for designing a VAR2CSA-based vaccine.

In a previous work, molecular analysis of VAR2CSA DBL3X sequence of placental isolates had suggested that distinct parasite variants preferentially infect pregnant women according to their parity status [20]. Among the 6 VAR2CSA DBL domains, antibodies raised against DBL3X and DBL5ε recombinant proteins are the most likely to cross-react with heterologous parasites including placental isolates from Tanzania [21,22] Moreover, previous studies in the same cohort showed that the women serum recognized preferentially the DBL5ε domain and that the level of DBL5ε antibodies was associated with a protection against the placental infection [7,23]. These accumulated observations motivated the need for analyzing the molecular variations occurring in this immunodominant domain of the VAR2CSA. Deciphering the parasite population dynamics occurring during pregnancy that result into segregation of genotypes according to woman parity or outcomes would be of particular interest in the development of an efficient vaccine against PAM.

This study presents parasite population dynamics in a cohort of pregnant women in Senegal. Genotypes of the parasites infecting pregnant women were determined by *msp2 *and microsatellites genotyping. The sequencing of the *var2csa DBL5ε + Id5 *(*Interdomain 5*) part and analysis of associated polymorphism was also performed from parasites collected at different time points of the survey.

## Methods

### Ethics statement

For the whole study, the human experimentation guidelines of both French and Senegalese health authorities were followed. Women included in the study were explained the nature of the project, and informed oral consent was obtained in the presence of an external and independent witness. The study design, the sampling protocol, as well as the way to collect informed consent were approved by the ethical committee of the Ministry of Health, Senegal. Notably verbal consent was considered valid at the time of study and recommended in this context of poorly literate study population.

### Study population

Pregnant women were enrolled in a cohort study between 30 July and 15 October 2001 in Thiadiaye, 130 km east from Dakar, Senegal. As previously described [23], 306 women pregnant for less than six months were enrolled if they were not infected with malaria parasites at that time. Women were followed by active and passive malaria detection through monthly antenatal care visits and through weekly home visits until delivery. During home visits women presenting with fever were referred to the emergency visit and blood samples were made for parasite detection. Women presenting with fever and a positive blood smear were given curative treatment with chloroquine, the drug recommended in Senegal at the time of study. Peripheral blood was collected every month after the woman was included in the study and during emergency visits. Blood spots on filter paper were stored for later DNA extraction and plasma was frozen. At delivery, both peripheral and placental bloods were also collected and processed as previously described [24].

Placental infection was observed in a total of 45 women at delivery by microscopy and/or PCR. Among these women, 32 also experienced at least one positive peripheral parasitaemia episode between enrolment and delivery. Eleven of those 32 women were primigravidae, five were secundigravidae and 16 were multigravidae (gravidity ranging from 3 to 9). For the 32 women with consecutive parasitaemia recorded, a total of 201 samples were obtained. From these, 108 were shown to be *P. falciparum *positive. Four to 9 samples (including placental samples) were available per woman, of which 2 to 8 were infected. The determination of the parasite genotype was made by combination of genotyping data of the *msp2 *gene and of five distinct microsatellites.

### DNA extraction, *P. falciparum *PCR detection and *msp2 *genotyping

DNA extraction from filter paper spots was done by Qiagen extraction for all molecular analyses. *Msp2 *genotyping was done as previously described [25]. Amplification of *msp2 *fragment was done with a 5' fluorescein-labelled primer: 5'-f GAA GGT AAT TAA AAC ATT GTC and a reverse non-fluorescent primer: 5'-GAG GGA TGT TGC TGC TCC ACA G. The PCR product was resolved by a capillary gel electrophoresis in an ABI Prism 3100 Genetic Analyser (Perkin Elmer Applied Biosystems). The results were analysed using GeneScan software (version 3.7; Applied Biosystems). The internal lane standard was labelled with TAMRA to distinguish PCR products from the standards and consisted of 28 bands ranging in size from 37 to 14,079 bp. The chromatogram generated for each PCR product allowed size identification of *msp2 *genotypes and their quantification through measures from the area under the curve of the corresponding peak.

### *Plasmodium falciparum *microsatellites genotyping

Five microsatellites were analysed: TAA81 (Genbank AF010510), TAA87 (Genbank AF010571), TAA60 (Genbank AF010556), PfPK2 (Genbank X63648) and ARA2 (Genbank X17484) [26-28].

Amplification of those 5 fragments was done with two multiplex PCR (one was optimized for TAA81 and TAA87, and the other for TAA60, ARA2 and PfPK2). The primers used were:

- for TAA81: the forward primer 5'-CATTTCACACAACACAGGATT-3' and the reverse primer 5'-FamGAAATAAGGGAAGGTGAGGA-3'.

- for TAA87: the forward primer 5'-ACATGTTCATATTACTCACCA-3' and the reverse primer 5'-FamCATTCAACCACCTAACAAC-3'.

- for TAA60: the forward primer 5'-GGTAAAAAAAGGAGGATAAAT-3' and the reverse primer 5'-NedAAGTAGTAACGATGTTGACAAA-3'.

- for ARA2: the forward primer 5'-TCCGCTTTGAGTATTATTA-3' and the reverse primer 5'-HexTTAAAAACCAGAATTATCTAA-3'.

- for PfPK2: the forward primer 5'-ATTCCTTTCATCGCTACTAC-3' and the reverse primer 5'-HexAAAGAAGGAACAAGCAGA-3'.

PCR products were resolved by capillary gel electrophoresis. The internal lane standard was labelled with ROX, and the 5'-primer used for the PCR was labelled with 6-carboxy-fluorescein, hexacloro-6-carboxy-fluorescein or trichloro-6-carboxy-fluorescein. The standards consisted of 28 bands ranging in size from 35 to 350 bp. Each genotype was characterized as described above.

### *Var2csa DBL5ε + Id5 *cloning and sequencing

The *var2csa *area spanning the *DBL5ε *and the hypervariable *Id5 *were amplified from genomic DNA using high fidelity enzyme (Phusion). The primers used for amplification were designed in the highly conserved blocks flanking the *DBL5ε+ Id5 *following alignments of the 18 *var2csa *sequences that were available online. Primer sequences are DBL5ε F: 5'-GTC ACC CCC GGG GAC AAT GCA ATA AAA GAT TAC and DBL5ε Rv: 5'-TAG GCA TTT GCG GCC GCC TTC AAG TTC AGC TGG AAT ATT. The PCR products were digested with XmaI and NotI for cloning into the baculovirus vector, pAcGP67-A [29]or were inserted into a topo TA cloning vector. A minimum of 5 clones per monoallellic isolate and up to 18 in polyallelic samples were sequenced to analyse multiple reads for one sequence and thus limit sequencing artefact. Sequencing was performed by GATC company [30] (Accession Numbers [Genbank: GQ476827-GQ476874, GQ476876-GQ476980]). Sequences generated were translated using the RevTrans webserver [31]. Only sequences without errors and stop codons were used for the analysis. Two sequences were considered as common *DBL5ε + Id5 *gene type if they had more than 96% sequence identities as previously described [32]. The final data set of *DBL5ε+ Id5 *sequences obtained from samples of the 32 pregnant women at different time points in pregnancy contained 172 sequences, fourty eight of these originated from placental isolates.

### Analysis of codon-based evolutionary models

Nucleotide sequences were aligned using the RevTrans server with a translated multiple alignment of corresponding sequences as input. The multiple alignment of nucleotide sequences was used for constructing a maximum likelihood phylogenetic tree using the program PAUP [33]. The maximum likelihood tree and nucleotide multiple alignment was used for detection of positively selected sites in *DBL5ε + Id5*, using the program codeml from the PAML package version 3.15 [34]. Four codon-based models were tested: M1, M2, M7 and M8, where M1 and M7 assume all dN/dS ratios to lie between 0 and 1 (no positive selection), and M2 and M8 assume an extra class of codons where dN/dS >1 (positive selection). Comparison of likelihood values for M1 and M2 or M7 and M8 were performed using a chi-square test. The Bayesian empirical Bayes procedure was used to estimate the significance of dN/dS ratios in the *DBL5ε + Id5 *[35]. Only codons with P < 0.05 in both models M2 and M8 were identified as positively selected sites. Positions corresponding to positively selected sites were identified in the DBL5ε structural model published earlier [36], and mapped using PyMol [33].

### Analysis of differential amino acids sequences

The translated multiple alignment generated with MAFFT was used for analysis of sequence differences between different groups of the data. In addition, two alternative sets of *DBL5ε + Id5 *sequences were included in the analysis: 21 sequences were from 11 isolates of cerebral malaria patients, and 19 sequences from 10 nulligravid infected women. The SigniSite method was used for identifying positions which have differing amino acids correlated to the ranking of sequences (Hoof *et al *Submitted). Analysis of sequences from PAM isolates according to birth weight of the children or parity of the women were also performed.

### Anti DBL5ε antibody level

The DBL5ε domain of a placental isolate obtained in a previous study in Senegal [3] was amplified, cloned, and the corresponding recombinant protein produced in baculovirus-infected SF9/Hi5 cells, and purified as described [23]. The recombinant protein was coated in 96-well plates, and the level of specific IgG in plasmas of the 32 women at different time points was measured by ELISA as previously described [37].

## Results

### Multiplicity of infection as determined by *msp2 *and microsatellites genotyping

Genotyping at polymorphic locus such as block 3 of the *msp2 *and the analysis of the 5 distinct microsatellites studied here allowed the appreciation of the parasite population structure for each recorded infection. Of the 108 infected blood spots analysed, complete genotyping data were obtained for 104 samples. During the follow up, the number of distinct malaria episodes recorded per woman ranged from 2 to 8 (1 malaria episode here correspond to the detection of *P. falciparum *in the woman blood sample). When the same parasite population (the same genotype) was found in successive samples of the women who did not receive treatment in between, this was not considered as a new malaria infection. It should be noted that many malaria parasite detections were performed retrospectively by PCR in women samples, meaning that treatment was not associated to each positive sample. The number of distinct parasite genotypes that have been successfully identified per sample ranged from 1 to 4, among the 2 to 8 positive parasitaemia that were detected throughout per woman during the follow up.

Based on *msp2 *and microsatellites genotyping and previous sequencing data available in the laboratory, the number of clones to be sequenced was arbitrarily defined as 5 clones in monoclonal samples and 18 in polyclonal samples.

Identical or almost identical *DBL5ε + Id5 *sequences from different women were rare. Actually, we could identify 64 *DBL5ε + Id5 *sequence types among different women based on a 96% sequence identity cut off, when applying a more stringent cut off of 99% we could identify 104 unique *DBL5ε + Id5 *sequence types. Common *DBL5ε + Id5 *types were found in 53 samples from different women while using the96% cut off, while common types were only found in 13 distinct isolates with a 99% cut off. This indicate low overlap in the *var2csa *gene sub-repertoires between parasite population from distinct isolates. In consecutive samples from the same woman it was possible to identify similar *DBL5ε + Id5 *types sharing a 100% sequence identity.

Fifty seven (55%) samples were monoallelic according to the genotyping (Table 1). Among the 104 samples, 54 presented with the same number of *msp2*/microsatellite genotypes and *DBL5ε *sequence types. 21 presented with a smaller number of *DBL5ε *sequence types than of parasite genotypes. These differences are probably related to the lower sensitivity of the cloning/sequencing technique, as compared to the optimized genotyping methods. Actually, Genescan detection of fluorescent fragments allows for detection of minor genotypes down to 2% of the population [25]. For 29 isolates, an unexpected bias in the *DBL5ε *+ Id5 sequence number compared to the genotype number was noted. This was unexpected considering the higher sensitivity in population structuring of *msp2*/microsatellite genotyping over the gene cloning and sequencing in general, and supports previous findings that some circulating *P. falciparum *parasites, like the HB3 strain, may harbour more than one *var2csa *copy in their genome [38,39].

**Table 1:** Comparison of the number of *DBL5ε/Id5 *sequences and of genotypes for the 104 isolates.

		*DBL5ε/Id5 *sequence types				
		
		1	2	3	4	Total
*Msp2 *and microsatellites genotypes	1	33	23	1	0	57
	2	13	18	4	1	36
	3	1	7	2	0	10
	4	0	0	0	1	1
	
	Total	47	48	7	2	104

### Dynamics of parasite populations during pregnancy

**Figure 1:**
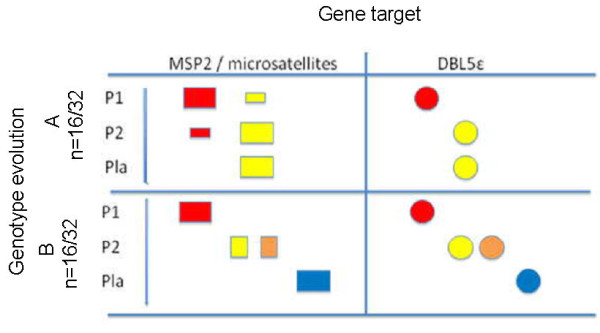
**Dynamics of parasite populations during pregnancy**. Two kinds of patterns (A and B) are described. P1, P2 represent positive parasitaemia from peripheral blood collected during the pregnancy, Pla represents placental blood collected at delivery. *msp2*/microsatellites genotypes are represented by squares. For one pattern, the various colors of the square represent a distinct *msp2*/microsatellite genotype. The different *DBL5ε+Id5 *sequence types are represented by different colored circles.

In the particular context of Senegal, where chloroquine was still recommended at the time of study, the overall analysis of parasite genotypes evolution at different time points during pregnancy shows two patterns according to *msp2 *and microsatellites polymorphisms. These two patterns were confirmed by analysis of variable blocks in the *DBL5ε + Id5 *sequencing (Figure 1):

- Pattern A: (n = 16): In this group of women, emergence of a specific genotype which can persist, and be found in the placenta at delivery is observed. The persisting genotypes are detected here as early as 69 days before delivery and in the following samples.

- Pattern B: (n = 16): In this group, several distinct infections or emerging genotypes are recorded during the follow up. Many samples from this group were *P. falciparum *negative suggesting that those women cleared their infection before being reinfected by novel parasite populations. The genotypes found in the placental blood at delivery have previously not been detected in the peripheral blood during the follow up.

No significant difference was seen when comparing means between consecutive samplings from pattern A and pattern B. The patterns A and B only show samplings with positive parasiteamias. Many samplings with no parasite detected were observed in pattern B suggesting clearance of previous infection hence the renewal of parasite populations observed in sub-sequent samplings of that patterns, as opposed to pattern A where no clearance was seen in consecutive samples.

The placental parasiteamia did not differ between the 2 patterns (p = 0.35, Mann Whitney, *U*-test). The women, belonging to the different patterns, differed neither in their parity status (p = 0.46, Mann Whitney, *U*-test ), the birth weight of the offsprings (p = 0.37, Mann Whitney, *U*-test), nor in the time of delivery (p = 0.41, Mann Whitney, *U*-test), the latter to consider the time of possible exposure to malaria transmission. The mean time elapsed between sampling during pregnancy and delivery did not significantly differ among women in pattern A and in pattern B. This suggests that the genotypes do not persist in group A because the time between samplings and delivery was shorter compared to group B.

### Impact of the parasite population structure

**Figure 2:**
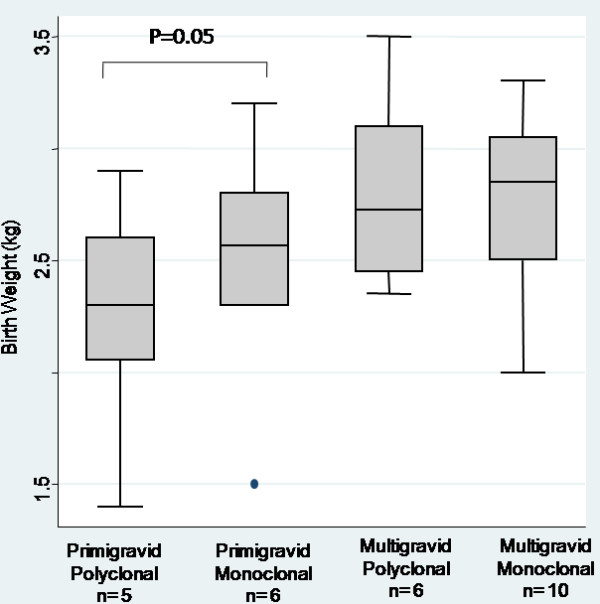
**Distribution of Birth Weight according to parity and to diversity of *Plasmodium falciparum *placental infection**. Diversity was determined by *msp2*/microsatellites genotyping.

The disappearance of the different genotype from the woman blood was not different when women received a curative dose of chloroquine or not during the follow up (p = 0.4, Mann Whitney, *U*-test), This could be explained by parasite resistance to chloroquine at the time of study that was later supported by the high prevalence of *pfcrt *K76T mutants (>70%) in this same cohort [40]. No relation between the complexity of infection and parasite density of the samples was found (p = 0.4; Mann Whitney *U*-test). However, birth weight of infants born from primigravid women differed according to the complexity of infection: primigravid women presenting with a monoallelic placental infection delivered babies with a higher birth weight than those from women harbouring more than one parasite genotypes (figure 2, p = 0.05, Mann Whitney *U*-test).

### Levels of anti-VAR2CSA-DBL5ε antibodies

When women were segregated on whether they harboured persistent/long lasting genotypes (pattern A), or not (pattern B) on Figure 1, the mean level of anti-VAR2CSA IgG was lower at enrolment in the former group (mean = 65.05 AU, standard deviation 27.62) as compared to the latter group (mean = 83.45 AU, standard deviation 16.56) (p < 0.05, Mann Whitney, *U*-test). At delivery, the mean levels of anti-VAR2CSA IgG were similar between the two patterns (p = 0.41, Mann Whitney): respectively 94.3AU (n = 15, standard deviation: 10.6) and 93.7 AU (n = 12, standard deviation 10.1).

### Sequence analysis of the *var2csa DBL5ε + Id5*

**Figure 3:**
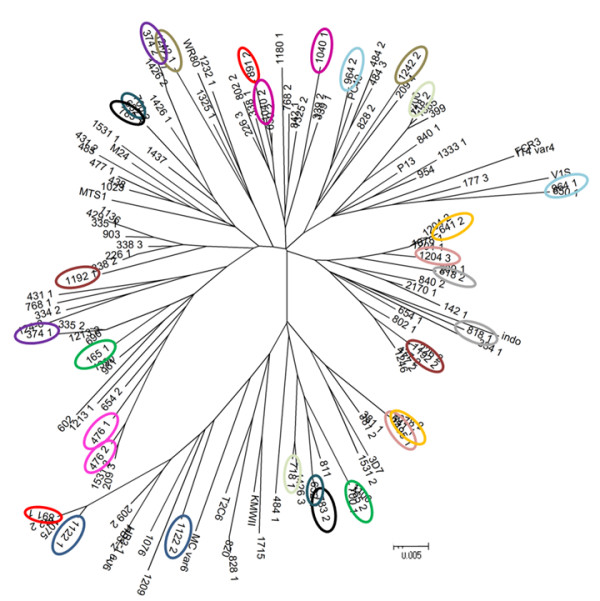
**Phylogenetic relationship between different VAR2CSA DBL5ε sequences**. Bayesian inference tree of 172 DBL5ε sequences from PAM isolates and 17 DBL5ε database sequences available from Genbank (MTS1, M24, WR80, PC40, P13, FCR3, IT4var4, V1 S, Indo, 3D7, KMWII, T2C6, Mcvar6, HB3-1, HB3-2, PC49, P13). Multiple and distinct DBL5ε sequences from monoallelic isolates are shown in colored circles.

To investigate the effect of parasite population dynamics on that of *var2csa *types, the *DBL5ε + Id5 *part of *var2csa *were sequenced from all isolates and 172 sequences were obtained from women. Multiple alignment of these sequences (Additional file 1) showed that the *var2csa *area corresponding to its *DBL5ε + Id5 *domains is relatively conserved with a pair-wise amino acids sequence identity within 75-100% (average of 85%). When only the *DBL5ε *is considered, the amino acid identity averaged 88%. Standard phylogenetic tree construction demonstrated that sequences from different geographic origins (obtained from GenBank) were scattered evenly in the tree indicating that sequences of *var2csa *from Senegal isolates do not differ at the *DBL5ε*+ *Id5 *area from those of the global repertoire (Figure 3), as previously observed on the DBL3X domain.

When focusing on the 2 distinct *DBL5ε *+ *Id5 *sequences found in some monoallelic isolates, the relationship between the 2 sequence types still appeared to be as distant as between any other different isolate.

### Identification of amino acid variation in the sequences of DBL5ε

**Figure 4:**
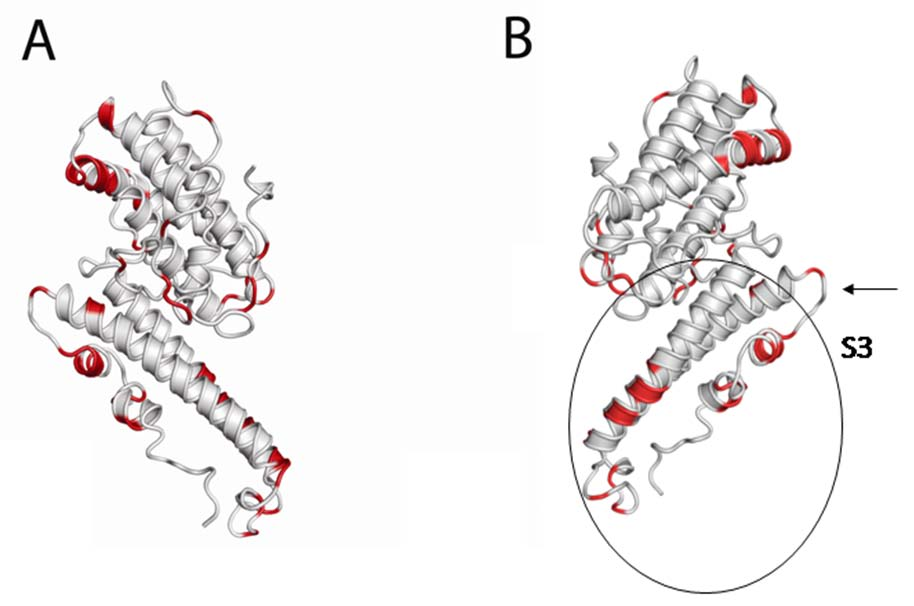
**DBL5ε variable positions**. A. Positions where all residues have a conservation of <70% in the alignment of 172 sequences from pregnant women are mapped in red on a DBL5ε structure model [36]. B. The same mapped positions as A, where the model is rotated 180 degrees, showing the variable sites mostly located in the S3 region of the DBL structure.

The variations in amino acid sequence are mostly located in variable blocks, in loop regions on surface exposed parts of helices, mostly in the S3 region (Figure 4), as previously reported for other VAR2CSA DBLs sequences [20].

**Figure 5:**
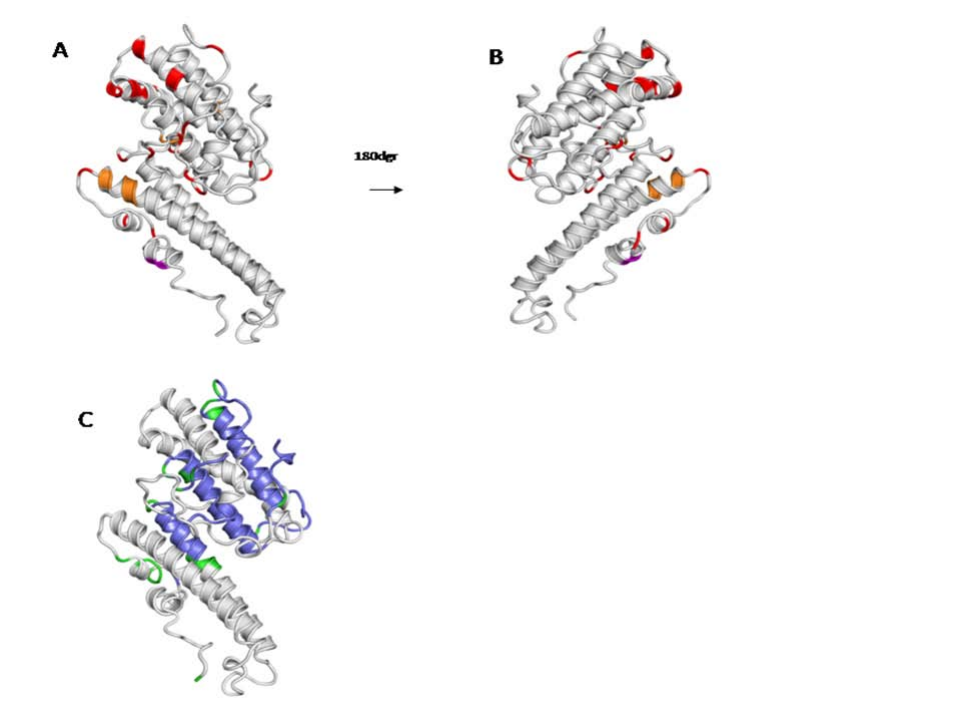
**DBL5ε conserved and immunogenic positions**. A. Positive selection sites mapped in the DBL5ε structure model are colored in red. In orange are shown sites which correspond to differential positions associated to parity and birth weight in the alignment of the 172 sequences from pregnant women. In purple is shown the position that is both under positive selection pressure and correlated with birth weight and parity. B. Similar to A, but rotated 180 degrees. C. Epitopes identified previously, in blue are shown the conserved residues, and in green, the variable residues, predicted to be targeted by surface reactive antibodies[51].

The calculated likelihood of model M1 (assuming no positive selection) was compared to M2 (assuming positive selection) and a chi-square test showed that the M2 model was significantly better than M1 (p < 0.001). Similarly, a test of the models M7 and M8 showed the model M8 assuming positive selection to fit the data better than M7 (p < 0.001). Positions identified to be under significantly positive selection in both models M2 and M8 are reported here. Thirty positions were found to be under positive selection or to correlate with parity and birth weight (see Figure 5A and 5B), and 22 of these are located in epitopes previously mapped in the model by Andersen [36] (see Figure 5C). Many of the positions under positive selection are located in proximity of regions, which vary in length (Additional File 1). This finding supports the previous finding, that most variability on the amino acid level is also located around gaps in the alignment. By mapping the positive selection sites in the model (Figure 5A and 5B), it can be seen that most positions under positive selection are located on the surface of both sides of the model and many are located in loops. Comparison of positions identified to be under positive selection with positions identified by SigniSite analysis, among sequences from cerebral malaria patients and from pregnant women, showed that only one position was redundant (Figure 5A and 5B). None of the residues identified by SigniSite to differ between parasites from nulli-gravid women and pregnant women were shown to be positively selected. Here, the search for such significant amino acid sites correlating with the ranking of sequences for clinical form, parity status, and birth weight showed that only a few amino acid positions were differing. These positions are 72, 152, 153, 290, 291, 294 and 320 in the alignment (Additional file 1). As the sequences are in general highly conserved, the distribution of amino acids is however not highly significant. Overall, these data show that there are only weak tendencies for differential patterns of amino acids in DBL5ε sequences from field isolates.

## Discussion

*Plasmodium falciparum *parasites exhibit extensive polymorphism, and natural infections often are composed of a mixture of genetically distinct parasite clones. The aim of this study was to determine the population dynamics of *P. falciparum *in pregnant women and investigate potential relation with malaria consequences during pregnancy.

Parasite genotyping at *msp2 *and 5 distinct microsatellites using a quantitative capillary-electrophoresis-based analysis of fluorescent fragments, allowed a better description of the parasite populations over time. The unexpected association bias in some isolates where more distinct *DBL5ε+ Id5 *sequence types were found as compared to the level of genetic diversity defined by *msp2*/microsatellites genotyping suggests that some circulating *P. falciparum *parasites can present with more than one *var2csa *gene copy in their genome. This was particularly highlighted in the 24 monoallelic samples where at least 2 types of *DBL5ε+Id5 *sequences were consistently found. This finding is not totally surprising as existence of 2 *var2csa *copies were already described in the genome of the laboratory strain HB3 and recently reported in several *P*. *falciparum *isolates [38]. Transcripts of such duplicated *var2csa *genes were also reported [39]. Although no relation was found here between these particular isolates with multiple *var2csa *copies and virulence, it will be of interest to investigate relationship between the parasite *var2csa *genome copy number and the pathogenesis of PAM in a larger cohort of dedicated study. Considering the present finding that more than one *var2csa *copy can co-exist in one parasite genome, the hypothesis for diversity generation suggested for other *var *genes could also be applicable to *var2csa *(UpsE genes) [41]. The copy number of *var2csa *in one genome still is limited and could explain why its diversity is not as enormous [18].

The multiplicity of a *P. falciparum *infection at a particular time-point is, indeed, a result of many factors, including epidemiology, clinical status of the host, intake of anti-malarial drugs, sequestration and degree of host immunity. Thus analysis from a single time-point sample presents limitations in revealing the infecting parasite populations. In pregnant host, immunity is an important factor as after successive pregnancies women acquire a high level of anti-VAR2CSA IgG, and a protection from low birth weight, placental infection and malaria anaemia [42]. Exploring the impact of the antibody level to VAR2CSA on clinical protection, and also on the *Plasmodium *genotype dynamics thus appeared as a necessity.

In a previous study on pregnant women living in a perennial transmission area, the diversity of placental infection was not related to parity or parasite density [43], while, in a seasonal transmission setting, polyallelic infections in the placenta were preferentially found in primigravidae [44]. Here, the complexity of the infection at delivery in the placenta, was associated to a lower infant birth weight in primigravidae. This effect was not found in multigravidae. Reduced risk of clinical malaria in children infected with multiple clones of *P. falciparum *is often reported [45,46]. The finding in this study rather points out the multiplicity of infection in the placenta as a potential risk factor to low birth weight in women having no or low immunity against placental parasites. One possible explanation for this virulence factor may be that in naïve hosts the survival of the parasite population is controlled by a competition occurring between the genotypes present leading to an augmentation of their virulence, whereas in immune hosts, such competition is limited by the specific non-sterilizing immunity, that allows the persistence of some genotypes without any clinical impact [47].

Analysis of the within-host dynamics of *Plasmodium *infection during pregnancy in this study allowed to visualize the picture of the parasite populations over the course of pregnancy in a context of drug inefficacy. Actually similar parasite genotypes were found in consecutive blood samples collected from women at different time points during pregnancy. The analysis clearly points that some parasite variants were able to persist over several weeks, and were found at delivery in the placenta while others were cleared. The role of anti-VAR2CSA antibodies in parasite clearance is suggested as women with low level of these antibodies early in pregnancy could not clear their infection. Different studies [11,20,48] suggest that parasites have different binding capacity or biological advantage that may promote their survival in distinct physiological environments. In the current longitudinal investigation, long lasting parasite genotype not only appeared to result from the inefficacy of chloroquine that was later proven [40], but also to low anti-VAR2CSA immunity at early pregnancy. This highlights a possible role of anti-VAR2CSA antibodies in parasite clearance and is in line with recent observations that high levels of antibodies to VSA-PAM in pregnant Malawian women correlated with malaria parasite clearance and less anaemia in a context of treatment failure [49]. As women with high anti-VAR2CSA IgG still could be infected by novel parasite genotypes with a distinct *var2csa *variant in their genome, it is possible that attaining the mature and protective immunity induced by VAR2CSA may require a gradual acquisition of antibody targeting a limited repertoire of variants.

PfEMP1 proteins are generally highly polymorphic and under significant antibody selection at the IE surface for diversification. This diversity was also described in *var2csa *in a previous study based on its DBL3x domain [20], demonstrating that *var2csa *could vary in sequence between primigravidae and multigravidae. Attempts in the current study of correlating *DBL5ε *sequence types to variables such as parity or birth weight might have been distorted by use of gDNA instead of cDNA as it now clearly appears that some parasites can harbor more than one *var2csa *gene copy in their genome. Although few significant sites under positive selection can be found in the amino acid sequence of the *DBL5ε*, a relatively high homology was noted among sequences from all field isolates studied. The contrast between this high level of conservation in this domain and the large diversity previously described for other non *var2csa var *gene [32], can explain why antibodies induced by DBL5ε recombinant proteins are cross-reactive to different placental isolates [22], and why serum from women from different origins are able to recognize the same placental isolates [4]. Conformation constraints within this domain seem to be important and necessary to the parasite, making this area of VAR2CSA an attractive target for induction of cross-reactive antibodies.

## Conclusions

Two patterns of *P. falciparum *dynamics in pregnant women are described in this study according to the anti-VAR2CSA IgG level of the women at enrolment. The correlation between low levels of anti-VAR2CSA antibodies and the presence of persisting parasite genotypes in the placenta at delivery suggests that the acquisition of a protective immunity in pregnant women is gradual. This protective immunity directed against VAR2CSA is acquired after only one or two pregnancies, suggesting that the target of antibodies must be relatively conserved. The DBL5ε domain is one of the immunodominant part of VAR2CSA [22,23,37,50,51]. It's sequence appears much more conserved than other non *var2csa var *genes, suggesting that this domain might be an interesting target for vaccine development aiming at inducing cross-reactive immunity.

Some parasite isolate may harbor more than one *var2csa *copy in their genome, making necessary further analysis on transcripts to better evaluate the impact of such variations in the *var2csa *variants fluctuation among pregnant women and in the acquisition of protective immunity. It is, therefore, of major importance to identify the few critical VAR2CSA variants, the combination of which can induce antibodies with broad reactivity against PAM parasite isolates.

## Competing interests

The authors declare that they have no competing interests.

## Authors' contributions

JG: participated in the conception of the research, the design of the study, the sample processing, the analysis and interpretation of the results, the literature search, and writing the manuscript. PA participated in the analysis and the interpretation of the results, and the writing of a part of the manuscript. CE participated in sample processing. SG participated in the critical review of the manuscript. NF participated in sample collection. OL participated in the critical review of the manuscript. PD participated in the concept of the study, found the resources needed, and participated in the critical review of the manuscript. NTN participated in the concept of the study, in the design and supervision of the project, contributed in finding the resources needed, participated in the analysis and interpretation of the results, the literature search and the writing of the manuscript.

All authors read and approved the final manuscript.

## Supplementary Material

Additional file 1 **Multiple Alignment of VAR2CSA DBL5ε sequences**. The DBL5ε sub-domains S1, S2, S3 and the interdomain Id5 are indicated. The sequences have been deposited in GenBank. Click here for file
