# Supplementary figures and images for: Plasmodium falciparum population dynamics in a cohort of pregnant women in Senegal
Source: Malar J. 2010 Jun 16;9:165. doi: 10.1186/1475-2875-9-165 (PMC2893538; doi:10.1186/1475-2875-9-165)

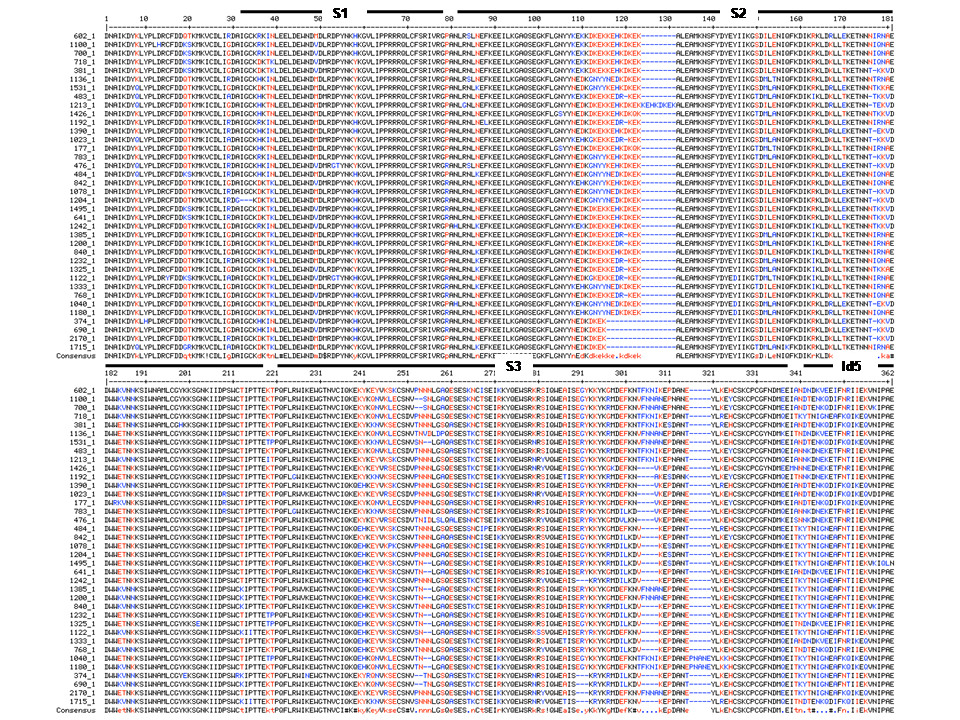

Supplement: Additional file 1 — Multiple Alignment of VAR2CSA DBL5ε sequences. The DBL5ε sub-domains S1, S2, S3 and the interdomain Id5 are indicated. The sequences have been deposited in GenBank. [file 1475-2875-9-165-S1.TIFF]
